# Commentary to the paper by Walter Dempsey and Peter McCullagh

**DOI:** 10.1007/s10985-018-9444-5

**Published:** 2018-07-18

**Authors:** Hans C. van Houwelingen

**Affiliations:** Department of Biomedical Data Sciences, Leiden University Medical Center, Leiden, The Netherlands

## Introduction

The authors are to be congratulated for a very interesting paper. They are also be thanked for recommending reading chapter 8 of our book on dynamic prediction (van Houwelingen and Putter 2012) The data in that chapter are extensively analyzed in the unpublished PhD thesis of Mark de Bruijne. One chapter is published as de Bruijne et al. (2001a). Unfortunately the chapter using the revival process never got published. A preprint (de Bruijne and van Houwelingen 2001b) is available.

I am pleased by the introduction of the concept of “stale measurement”. It is related to the concept of “ageing covariate” in section 5.3 of van Houwelingen and Putter (2012). In de Bruijne et al. (2001a) the concept “TEL(t) = time elapsed since last observation” is introduced as a tool to adjust for the staleness of observations. It is a nice feature of the revival approach that TEL(t) is inherently taken into account.

My main interest is the predictive use of the revival process. My comments arise from this preoccupation with prognosis. My plea for robustness in van Houwelingen (2014) arises from the need to validate prognostic models in new data or by cross-validation. Robustness is needed to ensure that the models are validation-proof. In the paper, the robustness of the revival model is explored in the supplementary material, but no attempt is made to check the robustness of the prediction model. Robustness of the implied prediction model is also an important issue in Rizopoulos et al. (2014) and Rizopoulos et al. (2017).

In this commentary I will focus on four issues: visualization of the data, more insight in the information carried by the revival process, validation of the implied prediction model and an alternative for the $P(T<\infty )=1$ assumption. I will use the CSL1 data and the standard model of section 6—exponential marginal survival with $\lambda _0=0.164$ and revival model based on the uncensored observations—to clarify my comments.

## Visualization

**Fig. 1:**
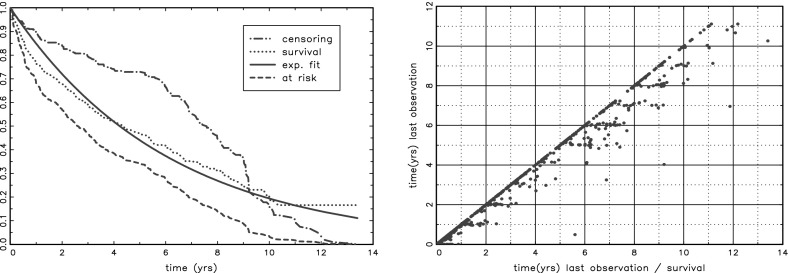
Survival etc

The two graphs in Fig. 1 are helping to get more insight in the data structure. The left panel shows the Kaplan–Meier estimates for the censoring function, the survival function with its exponential fit and the fraction still at risk. The high rate of early censoring is a bit unexpected, but its discussion is beyond the scope of this commentary. The interesting point for me is that $t=9$ appears to be the observation limit in this data. Only 43 patients carry information about what happens after $t=9$ and most of them (36) are censored. Anything said about what happens after $t=9$ is very speculative.

The right panel is an attempt to visualize how long patients are still followed up for survival after the last measurement. For each patient the difference between observed survival/censoring time and the time of the last observation can be found by the horizontal distance between the isolated dots and the dots on the $45^{\circ }$ line. One might wonder what happened to the patients with a wide gap between the last measurement and the survival time, but that issue is also beyond the scope of this commentary.

## More insight in the information carried by the revival model

**Fig. 2:**
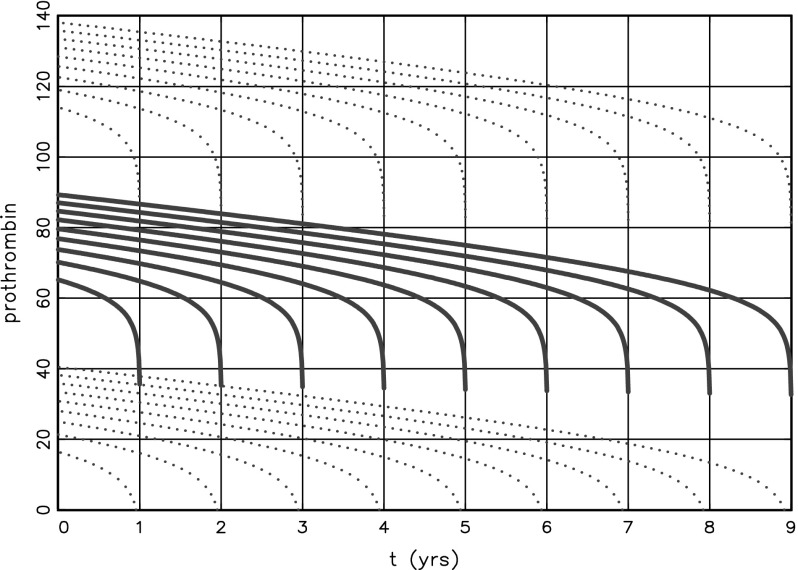
Insight in revival model

Figure 2 shows the expected value $\mu (s)$ of the revival process for $T=1,\ldots ,9$, presented in follow-up time t. The solid graphs show the curves for “Null Treatment” which can be seen as the expected value corrected for the additive treatment effect. The steep decrease near $t=T$ seems promising for the use of the revival process in dynamic prediction of survival. However, there is substantial variation in the data. The total variance computed from the three variance components in the model is 625, giving a standard deviation $\mathrm{sd}=25$. The tolerance regions $\mu \pm 2*sd$ are shown by the dotted lines. The large variation suggest that it would not be easy to infer the future T from the data available at time t.

If we ignore the uncertainty in the regression parameters we have a model *f*(*observations*|*T*). Given the observation history, inference on T can be made in a very classical way by computing the log-likelihood of the data. In the main paper such a log-likelihood is shown in the left panel of Figure 5 for one specific case. To get more insight a kind of landmark analysis was carried out, in which all 278 patients still at risk at $$t=2$$ are considered. The number of preceding observations varied from 2 to 9 with mode=4 .For each individual the log-likelihood *ll*(*T*) of the standard model is computed for survival time $$2 \le T \le 9$$. For each individual the location $$T_{max}$$ of the maximum is obtained together with a quasi $$\chi ^2 = 2 \cdot (ll_{max}-ll_{min})$$. If this $$\chi ^2 < 3.84 $$ and $$ T_{max}$$ is not on the boundary of the interval, the $$95\%$$ confidence region for *T* contains the whole interval [0,9]. A summary of the results is given in Table 1.

**Table 1:** Results of a classical maximum likelihood analysis at $t=2$

$\chi ^2$	$T_{max}=2$	$2<T_{max}<9$	$T_{max} \ge 9$	Total
0–1	61	43	94	198
1–2	11	4	39	54
2–3	4	3	8	15
3–4	0	0	4	4
4+	2	2	3	7
Total	78	52	148	278

Figure 2 helps to understand what is going on. If the last observation is quite low, the best fitting curve would be obtained by $$T_{max} < 2 $$. However, the patient is still alive at $$t=2$$, which moves the $$T_{max}$$ to 2. If some observations are quite high, that would be an indication for survival beyond $$T=9$$ and $$T_{max}$$ will end up at the right boundary. The situation is more subtle, because of the random patient effect, but it is clear that it will not be easy to predict *T* at $$t=2$$.

## Prediction model

To investigate the behavior of the implied prediction model, the landmarking approach is used. That means that for a fixed time-point $t_{LM}$ in the follow-up we consider all patients still at risk, obtain the predictive distribution obtained from standard model using the observations available at $t_{LM}$ and compare that with the actual survival data. For the sake of robustness a horizon $t_{hor}$ is fixed and it is investigated how well the (conditional) survival up to $t_{hor}$ can be predicted. Table 2 shows the results for $t_{LM}=1,2,\ldots ,7$ and $t_{hor}=t_{LM}+2$, n = the number at risk.

**Table 2:** Calibration of the standard model

$t_{LM}$	n	Dead	c		$H_{pred}$		$\alpha _{c}$		$\beta _{c}$		
			${\hat{c}}$	se	Mean	sd	${{\hat{\alpha }}}_c$	se	${{\hat{\beta }}}_c$	se	Z
1	332	70	0.79	0.09	0.32	0.07	$-$ 0.90	0.60	1.03	0.53	1.96
2	278	61	0.83	0.11	0.33	0.07	0.37	0.71	2.13	0.65	3.30
3	229	46	0.75	0.11	0.33	0.06	2.53	0.89	4.27	0.85	5.03
4	188	36	0.69	0.11	0.32	0.06	1.52	0.96	3.38	0.91	3.73
5	166	37	0.86	0.14	0.33	0.08	0.46	0.68	2.22	0.64	3.44
6	136	30	0.94	0.17	0.33	0.06	0.96	1.13	2.58	1.05	2.45
7	96	23	1.04	0.22	0.33	0.06	0.64	1.24	2.19	1.14	1.92

First a simple calibration of the marginal survival is obtained through the model $\lambda _{cal}=c \cdot \lambda _0$ applied on the patients at risk at $t_{LM}$ and administratively censored at $t_{hor}$. The table shows the estimate ${{\hat{c}}}$ and it standard error. The apparent need for this correction can already be seen from Fig. 1.

Next we consider the cumulative hazard $H_{pred}$ from $t_{LM}$ up to $t_{hor}$ as obtained from the standard model for each patient. This can be seen as a summary of the prognosis.The modeled conditional survival is

$$\begin{aligned} P(T > t_{hor}|T \le t_{LM}, H_{pred})=\exp (-H_{pred}) \end{aligned}$$

The standard deviation of $H_{pred}$ gives insight into the variation in prediction between the patients in the landmark data set. The performance of the model can be checked through the exponential calibration model

$$\begin{aligned} \ln (\lambda (t|H_{pred}))=\alpha _c+ \beta _c \cdot \ln (H_{pred}). \end{aligned}$$

The calibration of the conditional survival is perfect if $\beta _c=1$ and $\alpha _c=\ln (0.5)=-0.69$. My cautious conclusion is that the standard model is not well calibrated, but the predicted cumulative hazard might be a useful tool in landmark type models because of its significance as shown in the last column. The Weibull model might be better calibrated. but I did not check that.

## Alternative for the assumption $P(T<\infty )=1$

Personally, I am very hesitant about any modeling beyond the observation limit $t_{lim}$=9. In situations where patients might be “cured”, models with $P(T=\infty ) >0$ do make sense. The advantage of the semi-parametric Cox model is that does not make any statement about what happens after the last observation. My suggestion for an alternative approach is to define an observation limit $t_{lim}$, to censor all patients at this limit, to make a revival model for $t < t_{lim}$ using the uncensored data and for $t \le t_{lm}$ using all patients that survive up to $t_{lim}$ and to estimate the marginal survival by Kaplan–Meier. This approach does not need any imputation. Moreover, calibration can now be based on the Cox model as well.

The coefficients in the revival model are shown in Table 3.

**Table 3:** Regression coefficients for the standard model and the alternative

	Constant	Control	Prednisone	T	s	$\ln (s+\delta )$
All uncensored	63.47	2.49	13.56	1.74	$-$ 2.11	4.66
Uncensored $t<9$	63.35	2.28	13.40	1.56	$-$ 1.80	4.58
All $t \ge 9$	75.15	6.55	18.97	1.47	$-$ 1.84	$-$ 0.176

**Table 4:** Calibration of the alternative model

$t_{LM}$	c		$H_{pred}$		$\alpha _{c}$		$\beta _{c}$		$\beta _{Cox}$		
	${{\hat{c}}}$	se	Mean	sd	${{\hat{\alpha }}}_c$	se	${\hat{\beta }}_c$	se	${\hat{\beta }}_{Cox}$	se	Z
1	1.01	0.12	0.26	0.10	$-$ 1.16	0.45	0.65	0.32	0.66	0.32	2.09
2	1.00	0.13	0.27	0.11	$-$ 0.59	0.48	1.07	0.36	1.06	0.35	3.07
3	1.00	0.15	0.25	0.09	0.80	0.59	2.13	0.46	2.09	0.38	5.57
4	1.02	0.17	0.22	0.09	0.71	0.65	2.00	0.47	1.97	0.38	5.23
5	0.99	0.16	0.30	0.15	$-$ 0.66	0.45	1.06	0.37	1.07	0.34	3.16
6	0.97	0.18	0.31	0.16	$-$ 1.28	0.49	0.48	0.38	0.48	0.37	1.31
7	0.94	0.20	0.42	0.24	$-$ 0.95	0.35	0.91	0.37	0.94	0.33	2.84

Table 4 shows the findings of the landmark analysis for the alternative approach. The first observation is that the marginal Kaplan–Meier does not need any calibration because it is model free. The deviations from $\mathrm{c}=1$ are due to the discrete nature of the Kaplan–Meier. The second observation is that the “prediction tool” $H_{pred}$ shows more variation within and between landmark sets than in Table 2. Next, we see that the calibration through the exponential model is much better: the estimates ${\hat{\beta }}_c$ are much closer to one than in the standard model. Finally, we see that the calibration Cox model gives virtually the same $\beta$ as the exponential with standard errors that are marginally smaller.

## Conclusion

The revival approach can be a very useful tool for taking account of the observation time in prediction models. The comparison of different approaches to obtain prediction models using the calibration in the full data set as shown above might be optimistically biased. To avoid this optimism bias some form of cross-validation is needed, but that is beyond the scope of this commentary.
